# A qualitative study exploring hospital-based team dynamics in discharge planning for patients experiencing delayed care transitions in Ontario, Canada

**DOI:** 10.1186/s12913-022-08807-4

**Published:** 2022-12-03

**Authors:** Lauren Cadel, Jane Sandercock, Michelle Marcinow, Sara J. T. Guilcher, Kerry Kuluski

**Affiliations:** 1 Institute for Better Health, Trillium Health Partners, Mississauga, ON L5B1B8 Canada; 2 Leslie Dan Faculty of Pharmacy, University of Toronto, Toronto, ON M5S3M2 Canada; 3 McMaster University, School of Rehabilitation Science, Hamilton, Canada; 4 Institute of Health Policy, Management and Evaluation, University of Toronto, Toronto, ON M5T 3M7 Canada

**Keywords:** Qualitative research, Patient discharge, Delayed discharge, Patient transfer, Teamwork, Team dynamics

## Abstract

**Background:**

In attempt to improve continuity of patient care and reduce length of stay, hospitals have placed an increased focus on reducing delayed discharges through discharge planning. Several benefits and challenges to team-based approaches for discharge planning have been identified. Despite this, professional hierarchies and power dynamics are common challenges experienced by healthcare providers who are trying to work as a team when dealing with delayed discharges. The objective of this study was to explore what was working well with formal care team-based discharge processes, as well as challenges experienced, in order to outline how teams can function to better support transitions for patients experiencing a delayed discharge.

**Methods:**

We conducted a descriptive qualitative study with hospital-based healthcare providers, managers and organizational leaders who had experience with delayed discharges. Participants were recruited from two diverse health regions in Ontario, Canada. In-depth, semi-structured interviews were conducted in-person, by telephone or teleconference between December 2019 and October 2020. All interviews were recorded and transcribed. A codebook was developed by the research team and applied to all transcripts. Data were analyzed inductively, as well as deductively through directed content analysis.

**Results:**

We organized our findings into three main categories – (1) collaboration with physicians makes a difference; (2) leadership should meaningfully engage with frontline providers and (3) partnerships across sectors are critical. Regular physician engagement, as equal members of the team, was recommended to improve consistent communication, relationship building between providers, accessibility, and in-person communication. Participants highlighted the need for a dedicated senior leader who ensured members of the team were treated as equals and advocated for the team. Improved partnerships across sectors included the enhanced integration of community-based providers into discharge planning by placing more focus on collaborative practice, combined discharge planning meetings, and having embedded and physically accessible care coordinators in the hospital.

**Conclusions:**

Team-based approaches for delayed discharge can offer benefits. However, to optimize how teams function in supporting these processes, it is important to consistently collaborate with physicians, ensure senior leadership engage with and seek feedback from frontline providers through co-design, and actively integrate the community sector in discharge planning.

## Background

Hospitals are increasingly focused on discharge planning in an attempt to improve hospital throughput, reduce length of stay and costs and enhance patient continuity of care [1, 2]. Delayed care transitions, often referred to as delayed hospital discharge or alternate level of care (ALC) in Canada, occur when a patient is occupying an acute care bed, but does not require the acute medical intensity of resources (i.e. is medically fit for discharge) [3]. Acute care teams can struggle to find the best discharge location for these patients especially for those with more medical and social complexity, often due to a lack of appropriate infrastructure in the community. For example, the inability to access long-term care homes or supportive housing [4–6]. The continued care for these patients while in hospital, as well as arranging an appropriate discharge are challenging because services in-hospital are often reduced or stopped entirely, leading to physical and cognitive deconditioning [7]. This can impact where patients can be transitioned to, especially if they lack family support. The financial situation of patients and their families can cause challenges and limit the possible retirement or long-term care homes to which they can transition [8]. Long wait lists for home and community services, such as physical and occupational therapy and personal support, can also cause challenges for patients who require such supports but cannot be adequately arranged. In addition to the difficulties experienced by acute care teams, patients and families have described frustrations with delayed discharges, including: uncertainty around their care journey, mental and physical deterioration, lack of engagement in decision-making, the inability to advocate for themselves, lack of services, and poor communication [9, 10].

These complex delayed hospital discharges require effective communication within hospitals and community organizations [11, 12]. Given this, team-based approaches, including integrated discharge teams and multidisciplinary rounds, have been recommended to improve the discharge planning process [13–15]. Ideally, patients and families are at the core of hospital care teams. In our previous research, patients with a delayed discharge and their caregivers have noted that improved communication amongst members of their care team is important and could contribute to better care transitions [10]. Hospital care teams also consist of physicians, nurses, occupational and physical therapists, social workers, pharmacists, discharge planners (who are often employed primarily as social workers, nurses or occupational therapists) and community care coordinators [16, 17]. Many providers on care teams play an active role in the discharge planning process, with the ultimate organizational goals of working collaboratively to ensure timely discharges, facilitating coordination of care, improving patient outcomes and reducing readmissions [2, 16]. Optimizing teamwork for discharge planning requires understanding how individuals work together to share perceptions, knowledge, experiences, and expertise between all members of the care team (also known as collective competence) [18, 19]. Some team-based approaches for discharge processes have demonstrated positive outcomes on achieving these aforementioned goals, including reduced length of stay and lower rates of readmission [14, 15, 20]. However, a number of challenges have also been identified with team-based discharge planning in acute care, including: managing professional hierarchies, dealing with conflict, understanding roles and responsibilities of other professions and handling communication breakdowns [21–24].

While several benefits and challenges with team-based approaches for discharge planning have been noted, little is known about how hospital-based healthcare providers on formal care teams function when dealing with the complexity of delayed care transitions. Therefore, we sought to explore what was working well with formal care team-based discharge processes, as well as challenges experienced, in order to outline how teams in hospitals can function to better support transitions for patients experiencing a delayed discharge.

## Methods

### Study design and theoretical orientation

A qualitative study was conducted to explore how hospitals in Ontario, Canada were implementing a provincial tool-kit and best practices for delayed discharge [25]. The tool-kit includes strategies for avoiding ALC designations and managing them once they occur [25]. These leading practices provide guidance on how to address delayed discharges through targeted efforts such as assigned estimated day of discharge for all patients, escalation processes, early discharge planning, staff and physician empowerment, identifying and reducing high-risk patients, outlining expectations for substitute decision makers, and physician and senior team engagement [25]. These leading practices are contingent on teams functioning optimally to support care transitions. Our research presents an in-depth analysis of the perceptions and experiences of key stakeholders (hospital-based providers, managers and organizational leaders) regarding challenges and potential solutions of team-based approaches to support delayed hospital transitions. While we acknowledge that patients and caregivers should be considered core members of the care team, the purpose of this paper was to shine a light specifically on individuals working within hospitals and how they function to support care transitions for patients and families; thus, they are the focus of this paper. The study followed an interpretive description approach in order to generate knowledge and insights that can be applied to clinical practice [26, 27]. The Standards for Reporting Qualitative Research were followed [28].

### Setting and participants

Two hospital networks within two diverse health regions (urban and rural) in Ontario, Canada served as the main sites for recruitment. The two hospital networks were chosen based on their implementation of the tool-kit and best practices for delayed discharge [25], as well as their differing geographical locations and rates of delayed discharges. A purposive sampling strategy was used to recruit key stakeholders, including providers, managers, and organizational leaders, who had experience with delayed discharges [29]. All participants were required to be over the age of 18, English-speaking and either working with patients experiencing a delayed discharge or involved in the implementation of strategies targeting delayed discharges. Initial participants were recruited through the research teams’ professional contacts (by email or telephone) and recruitment efforts were expanded through the use of snowball sampling. The number of participants who chose not to take part in the study was not tracked and no participants dropped out of the study.

### Data collection

Data were collected through one-on-one, semi-structured interviews from December 2019 to October 2020. The interview guide was developed by the research team and sought to explore experiences related to strategies targeting delayed discharges. We used the provincial tool-kit and best practices for delayed discharge to help guide some of the interview topics. More specifically, the interviews probed the participants to expand on their role and organizational contexts as they related to delayed discharge, barriers and facilitators to addressing delayed discharge (organization priorities, accountability, challenges and success stories), strategies in place or needed to prevent and manage delayed discharge (incentives, resources), and outcomes related to these strategies. Two trained members of the research team (MM, PhD; JS, MSc/OT) were responsible for data collection, with overall supervision and mentorship of the Principal Investigators (KK, MSW, PhD; SJTG, PT, PhD). The interviews were conducted in-person or virtually (e.g. by phone or Zoom) depending on the participants’ preference and lasted between 30 and 90 min in length. All interviews were audio-recorded and transcribed verbatim for analysis. Pseudonyms were applied to each participant and used to present quotes in the results.

### Data analysis

Data analysis occurred concurrently with data collection until data saturation was achieved [30]. Using a directed content analysis approach, the interviews were analyzed inductively and deductively (using the Alternate Level of Care Avoidance Leading Practices Framework [25]) [31]. As the interviews were conducted and transcribed, the research team met weekly to discuss the key concepts and ideas from the transcripts. These concepts and ideas served as the foundation of a preliminary codebook. Three members of the research team (LC, MM, JS) applied the preliminary codebook to a subset of transcripts to test their coding agreement (96%) and to ensure the codebook was comprehensive. The team met to discuss the preliminary coding and coders’ agreement, following which minor revisions were made to finalize the codebook. The final codebook was applied to all transcripts and contained 20 codes. These codes related to hospital/region characteristics, strategies in place or needed to address delayed discharge, factors impacting delayed discharge, stigma, power, tensions, professional education, politics, teamwork/interprofessional collaboration, as well as descriptions, and examples of each code. NVivo12 was used to organize the transcripts and perform the coding comparisons.

In order to gain a more in-depth understanding of the team-based approaches used during delayed care transitions and challenges with team functioning, code reports for teamwork and interprofessional collaboration and tensions were created and reviewed by members of the research team. In discussions with the research team, these codes were chosen to create code reports based on how they were defined in the codebook and the potential for them to contain data relating to the main objective (team-based discharge processes). Microsoft Excel was used to review, compare, and contrast these code reports. Overarching concepts and key ideas were discussed by team members (LC, KK, MM), merged, and named to become the themes presented in the results.

### Ethics

This study received ethics approval from the Research Ethics Boards at the University of Toronto (#00,038,126) and the two hospital sites (#961 and #19–046). All participants provided written consent prior to participation in their interview and agreed to have excerpts from their interviews published. All research was conducted in accordance with these organizational regulations and guidelines.

## Results

A total of 30 individuals participated in this study, including 17 from the urban health region and 13 from the rural health region. The majority of participants were non-physicians and based in-hospital; however, some were based at community sites affiliated with the hospital networks. Common roles of participants included: discharge planners, social workers, clinical and project managers, physicians, and team leads. The findings from the two regions were similar and thus, are presented together. We organized our findings into three main categories: (1) Collaboration with physicians makes a difference; (2) Leadership should meaningfully engage with frontline providers and (3) Partnerships across sectors are critical (see Table 1).

**Table 1:** What’s working well with team-based discharge processes?

Category	Working Well – Recommendations to Continue Doing
Collaboration with physicians makes a difference	• Regular physician engagement with team (as equal partners), including consulting with the broader team before transition decisions are made
	• Consistent communication between discharge planning team, patients and families
	• Relationship building between physicians and other providers
	• Physically accessible physicians
	• In-person communication (compared to email)
Leadership meaningfully engage with frontline providers	• Dedicated and visible senior leader who advocates on behalf of team
	• Promotion of equality amongst team (ensuring members of the team treated as equals)
Partnerships across sectors are critical	• Focus on collaboration across sectors
	• Discharge planning meetings (including hospital and community sectors)
	• Embedded care coordinators (on hospital units)
	• Physically accessible care coordinators

### Collaboration with physicians makes a difference

Physicians were described as powerful members of care teams, having a large influence on team dynamics and overall team functioning (how individuals interacted, communicated and worked together). Participants described that physicians who viewed themselves as solely responsible for discharge planning decisions often made decisions unilaterally without communicating or consulting with other team members. Other non-physician providers described feeling undermined when not consulted on discharge decisions, which created tensions within teams, as a discharge planner described:

*… what you don’t want to do is have the physician say [to the patient] – which does happen – “Well you can stay in the hospital.” Well, you may not know everything that’s behind the scenes or how the patient is doing, so talk to the team even before you [designate] the patients [with delayed discharge]. So that’s what we’ve been trying to do is get the physician to be involved with the team; don’t make unilateral decisions. (Farah, Discharge Planner)*

Unilateral decision-making had the potential to lead to miscommunication and ill-informed plans before and after designating a patient with a delayed discharge (not all members involved or aware of decisions), which in turn, created tensions between physicians and other providers, especially discharge planners. Some providers left out of discharge planning described feeling unprepared when physicians suddenly designated patients with a delayed discharge, needing to quickly organize supports to manage the change in the care plan and answer patients’ and families’ questions:

*… the physician sort of does their piece and then it’s write the ALC [delayed discharge] order, over to you to figure it out. And then the team is sort of rushing around saying, what am I doing, and the family has medical questions, and it’s I think a back and forth, and it doesn’t appear unified even to the patient and the family. I think here it’s sort of very disjointed. (Iris, Response Team)*

When the care team was not on the same page, or sharing consistent information, participants discussed how patients and families were at risk of becoming confused and at times, began to lack trust in the teams’ recommendations. A manager described the importance of clear communication and disseminating consistent information between team members and with patients and families:

*I just think we need to be really consistent in our messaging to patients and families… we need to make sure that everybody is delivering the same message… this is the message that we’re giving to patients and families and the patients and families are being told [something different] by the doc, like totally separate or right in the meeting even, saying no, no they can’t go home, they need 24/7. So that completely undermines the entire team. And the message. (Chloe, Manager)*

While the assertion of authority by physicians had the potential to negatively impact team dynamics through feeling devalued and creating uncertainty, team members acknowledged and appreciated, that patient safety was a primary concern of physicians. There was a perception among participants that physicians held the most responsibility regarding a patients’ medical needs. Therefore, they understood that physicians’ hesitancy to write discharge orders may have been due to a heightened sense of accountability, particularly if the patients’ future care needs were uncertain (e.g. number of required home care hours, ability to transition to pre-hospitalization residence, support from unpaid caregivers):

*If it truly is unsafe and a physician is not willing to write discharge order, they [the patient] will stay here…” (Nora, Clinical Manager)*

In contrast to these challenges, non-physician participants described that physicians who regularly engaged with the team and acted as equal partners in the discharge planning processes facilitated more positive collaboration, ultimately resulting in reduced confusion for patients, families and other team members, improved planning and enhanced team dynamics. Consistent communication also promoted relationship building between staff and created a foundation of trust, which were fundamental to engaging physicians as a core component of the care team and working through difficult cases together. Establishing a culture that promoted positive interactions and equality amongst physicians and the rest of the patients’ care team further enhanced perceptions of working together:

*I think regular communication. We have a fantastic team that actually is very helpful, so we communicate on a daily basis. We work together. We help each other out in difficult situations. (Monica, Social Worker)*

Daily rounds were used by the care team to discuss care plans and develop relationships. Since many physicians did not participate in these daily rounds because of timing conflicts and not being physically situated on the unit, more experienced discharge planners described seeking out physicians on the unit in order to share information and attempt to foster a collaborative culture. As positive relationships between physicians and discharge planners were developed, communication and collaboration around discharge planning processes were described to have improved:

*If I see the doctors on the unit, I go and have a conversation right off the bat or they’ll come and find me as well because I’ve got that relationship with them. And some of them are very good. They will come into the team room, they’ll talk with all of us or they’ll come and find me in particular and say, “Hey, this is what I’m thinking.”… and the new doctors that come in, I make a point of having conversations with them, building that [relationship] so that I can do that with them. (Becca, Discharge Planner)*

The ability to seek out physicians and other team members on units was facilitated by their physical location and proximity. Hospitals, as well as units within the hospitals, had different set-ups and location/organization of providers; however, participants noted that the ability to access team members and communicate in-person, rather than by email, improved collaboration and teamwork. For example, a discharge planner compared how physical office space located in close proximity to the unit facilitated easier communication amongst staff and physicians:

*I think it’s just the culture and how it operates… that it’s more collaborative, the docs work [comparing hospital sites*]*. Here it’s just even our team. Over there [other hospital site] basically every – I think besides emerg, all the social workers, discharge planners, they sit either in the – on the unit or right outside of it. They’re literally, like outside the doors. Whereas our team [at other hospital site] is spread out all over the place, the doctors [are] all over the place. There’s not enough computers, so we communicate here, generally, like everybody, right, the doctors and everybody, by email. (Gina, Discharge Planner)*

### Leadership should meaningfully engage with frontline providers

Frontline providers (non-physicians) appreciated being asked for feedback on initiatives and programs, especially when specific to improving discharging planning processes. However, when their feedback was not integrated by management (clinical/team leads) they felt undervalued. This often led to a lack of trust and strained relationships with management:

*So [the escalation pathway] was created by frontline individuals for the people who are going to be using it… But the one that was finalized and came out had kind of lost all of what was necessary to actually make it a functional document. And I kind of seemed to see that happen with a lot of things, where you hear there will be focus groups, you'll hear that we're going to listen to the frontline this time, but then what actually comes out a lot of times I don't see any of the feedback that was provided in it. (Pat, Discharge Planner)*

Leadership approaches that were perceived by staff as disrespectful, threatening or not open to staff input or ideas further damaged relationships and trust between frontline providers and management. At times, negative leadership styles created a unit culture that was described as not being conducive to providing input or improving discharge processes:

*People will not talk anymore because of all the issues that the [discharge planning] group has had… We’ve had a few really rough meetings and nobody wants to speak… I’m a vow of silence right now. I will not give my opinion anymore because you get labelled. (Becca, Discharge Planner)*

While a disconnect between frontline providers and management resulted in tensions and poor team dynamics, having a senior leader who was dedicated to addressing delayed discharges enhanced communication between frontline providers, senior management and community partners. This ultimately contributed to improved relationships and team functioning:

*I mean, [Lily’s] role as a senior transitions lead has also been very good. She emails us on a regular basis, keeping us up to date with what’s going on, what’s new, what resources we can tap into. (Monica, Social Worker)*

A leader who advocated for their team, ensured members of the team were treated as equals and was invested in improving processes around discharge delays created an environment in which collaboration and relationships could thrive. As one physician described:

*… and I advocate for my team, I will never advocate at the expense of any of the interprofessional team because they’re just as important as us. I really try and remove any hierarchy… I really try to equalize everybody. (Devon, Physician)*

### Partnerships across sectors are critical

The community sector (individuals employed by community agencies and community-based organizations) was an important piece of the discharge planning process as described by both hospital and community staff. Despite being an essential component of a patients’ care journey and discharge process, community organizations were not well integrated into the care team, which prevented smooth transitions and coordination of care. Hospital staff described that, at times, community organizations were resistant to changes around discharge processes and new cross-sectoral initiatives. For example, some discharge planners explained how community organizations did not want to adapt their usual discharge planning processes, even if it meant individuals experiencing delays could be discharged. This created tensions between hospital and community sectors, which further impacted relationships and team dynamics, as hospital staff felt isolated and unsupported by team members outside of the hospital. When individuals did not feel well supported by community organizations (limited engagement or desire to work together), relationships and future collaboration were negatively impacted. A discharge planner described challenges working with individuals from the health region, explaining that her ‘outside the box’ ideas for transitioning patients home were often shut down, limiting cross-sectoral collaboration. She also discussed the need to form a true partnership with the community sector to improve issues with delayed transitions.

*Although we are supposed to have partnership on paper, it’s not necessarily the case. Sometimes it’s very difficult working with the [health region]… So I think really the [health region] has to [be] brought on board and really become a partner in this [delayed discharge] issue that we have in the hospital. (Farah, Discharge Planner)*

With the perception of limited willingness from community organizations to adapt, hospital staff often felt restricted by factors beyond their control. This included having to keep patients in hospital because the community could not provide enough support, or community organizations were not accepting discharges on weekends. A discharge planner described how poor communication and misaligned procedures between the hospital and community affected discharge processes:

*… multiple times we've come into conflict with the [health region] saying, we have a patient who's ready for discharge, he needs these services, doctor says he's safe, and the [heath region which controls access to community health care resources] would say, we don't think they're safe to go home, and would effectively block a discharge, or delay a discharge. (Pat, Discharge Planner)*

Despite some challenges with teamwork across sectors, placing an increased focus on collaboration with the community improved communication, partnerships, relationships, access to programs and services, and problem-solving, particularly for patients with complex care needs:

*I find the working relationship right now is much more collaborative, it’s a lot more positive than what it has been in the past years. (Heather, Health Region Coordinator)*

Hospital staff described that improved collaboration and working relationships between individuals from hospital and community organizations were facilitated through discharge planning meetings. During these meetings, staff from both the hospital and the community worked together to develop a plan for patients experiencing a delayed discharge based on the supports they required for a safe discharge:

*We do meet with the [health region] once a week… meetings where we get together and we talk about our ALC [delayed discharge] patients… we are trying to work collaboratively with the [health region] and where we’ve got some great supports… the discharge planners come in and we present the case and then we try to develop solutions and try to develop timelines if the patient is [designated] ALC [delayed discharge] to try to get things moving along. (Farah, Discharge Planner)*

Team dynamics between the hospital and community sectors further benefitted from an embedded care coordinator role on the hospital unit. The embedded care coordinator was employed by the community sector, but was situated on a unit within the hospital. Care coordinators were not embedded on all units, but of those with one, participants highlighted their immense benefits on cross-sectoral partnerships and discharge planning for patients experiencing a delayed discharge. Embedded care coordinators were also physically accessible, improving communication and positively impacting team functioning and overall collaboration.

*We work very closely [with homecare] – like my office is right across the hall from the cubicle for homecare. So we have worked really closely with them trying to develop a relationship to be a collaborative one where we – if they have issues that they’re encountering on getting a patient home, then they can engage us, or vice versa. So, we’ve worked really hard on that to be all thinking the same thing. (Shae, Transitions Lead)*

## Discussion

This qualitative study explored how healthcare providers on formal care teams functioned when dealing with the complexity of delayed care transitions, and more specifically, what was working well and the challenges experienced. Based on our participants interviewed, our findings are most reflective of non-physician and hospital-based providers, not community based providers, thus it is important to interpret these findings with those missing perspectives in mind.

Understanding current challenges, tensions and opportunities allowed us to highlight key areas of improvement. Our findings map onto three main recommendations on how care teams can better function to support delayed transitions: (1) collaborating with physicians; (2) engaging with and strengthening the ties between frontline providers and leadership; and (3) partnering across sectors. Further to these recommendations, we discuss the importance of addressing and improving collective competence, engaging authentically and improving integrated care.

We found that regular engagement between physicians and other healthcare providers contributed to more positive communication, collaboration and relationships for members involved in discharge planning. This engagement appeared to reduce confusion for patients, families and other members of the care team regarding the patient’s discharge plan. Team culture, where each member of the team feels equally valued, was identified as an important factor. Communication and interpersonal relationships have been identified as precursors to physician engagement [32]. Communication should be transparent and bidirectional between physicians, other members of the care team, patients and families [32–34]. In terms of relationships, it is critical that both physicians and other team members feel valued, supported and respected [34–36]. For example, a qualitative study conducted by Baxter and colleagues explored factors to successful care transitions within high performing teams and identified knowing each other as a key facilitator to a safe transition in care [36]. More specifically, participants (doctors, nurses, healthcare assistants, receptionists, administrators, allied health professionals, discharge planners/coordinators, community nurses) noted the importance of feeling valued and listened to, building relationships across sectors or boundaries, and trusting one another. Establishing an organizational culture that supports equal treatment and value amongst team members involved in discharge processes is important. Interdisciplinary care for delayed discharges should include physicians as an integrated part of the patient’s care team. However, identifying how to structure a team and carry out organizational processes while ensuring that all team members, including patients and families, feel heard and valued is an area that requires additional work.

We also noted the importance of ongoing engagement from senior leadership with frontline providers. Frontline providers offered a unique perspective on challenges and opportunities as they related to improving delayed discharges because they had a front row seat to everyday processes. Interactions were negatively impacted when it was perceived that management did not seek or integrate feedback from frontline staff on processes or programs, often leading to strained relationships between frontline providers and senior leadership. Based on this finding and to avoid perceptions of tokenism, we recommend that programs, processes, initiatives, etc. implemented on the frontlines are co-developed in partnership with frontline staff, including a feedback mechanism. In terms of feedback integration, providers should receive a detailed explanation of how their feedback was used, or not used, and why. For example, if providers’ ideas could not be addressed or implemented for specific reasons, then this information should be relayed to improve transparency. Aligning with principles of patient engagement, this feedback mechanism can contribute to better relationships and teamwork through increased transparency and feelings of being valued [37]. It is important that an organizational culture ensures members of the team are treated as equals and distributed leadership is established to better facilitate the engagement of frontline providers with senior leaders [38].

The creation and maintenance of partnerships across sectors (integrated care) was our third recommendation for improving team functioning to support delayed transitions. While a multitude of definitions exist for integrated care, it can be understood as the continuum of health and social care (prevention, diagnosis, treatment) across sectors, with the goals of eliminating fragmented services and improving the quality of care delivery [39]. Integrated care has been previously recommended for patients experiencing a delayed discharge [40–42]; however, limited models exist for this population [41]. Given that the majority of patients who experience a delayed discharge are older adults with complex health or social needs [40], integrated care guidelines or models from other populations may also be applicable to those with delayed transitions. For example, an integrated care intervention was implemented among vulnerable, community-dwelling older adults in Montreal, Canada [43]. The multidisciplinary teams were community-based and responsible for completing geriatric and interdisciplinary assessments (e.g. falls, dementia, nutrition, depression, medication, vaccination status, diagnoses) in collaboration with the patients’ family physician. The multidisciplinary teams had case managers to ensure patients had the appropriate resources and to improve continuity of care across sectors. Despite not being designed and applied specifically for individuals experiencing a delayed discharge, this intervention resulted in a significant reduction in alternate level of care days (delayed discharges). Integrated care has resulted in a number of other positive outcomes including improved patient satisfaction, quality of care and access to care [44]. However, more research is needed to design standardized integrated care approaches in the Canadian setting for persons experiencing delayed transitions in care.

While we have identified areas of improvement, we extend our findings by exploring a key concept that we feel underlies the success of team-based approaches for improving delayed discharges: collective competence. Collective competence can be defined as, “the distributed capacity of a system, an evolving, relational phenomenon that emerges from the resources and constraints of particular contexts” (page S19) [45]. While each profession involved in the delivery or planning of care may be doing their job competently, the collective care provided by a team is not giving patients and families the results they want and/or expect. Collective competence is centred on how individuals interact and share their knowledge, experiences and perceptions and involves all members of the care team, including patients, families and the setting in which the interactions occur [18, 19]. It requires that all individuals have an understanding of their roles and responsibilities as they relate to each patient’s unique situation [19]. This concept reinforces that unless we prioritize and build ways to better communicate and collaborate, as a system, we may not be successfully integrating or providing competent care. Integrating collective competence in practice requires interprofessional education, observation and debriefing and ongoing commitment to change [19]. Building on the recommendations described above, organizations should place focus on improving the collective competence of their care teams that support patients with delayed discharges.

## Limitations

There are a few limitations of this research to report. Understanding patient and family perspectives on discharge processes and experiences with care transitions is important, and our team has explored this in our previous work [7, 46, 47]. Therefore, this research focused on care providers. We did not compare perspectives across participant groups, so we did not aim for data saturation of findings within types of providers. The majority of our interviews were conducted with hospital-based healthcare providers, but we had limited perspectives from physicians. In order to better understand the tensions across sectors in a more comprehensive way, future research should further explore the perspectives of physicians and community-based providers. Previous research has also noted the importance of cross sectoral team functioning [36]. Also, given that the main focus of the interviews was not to understand team dynamics, it is possible that if participants were specifically probed on these topics additional insights may have been provided. We also collected data in-person, by telephone and Zoom. We began data collection with in-person interviews prior to the COVID-19 pandemic. However, when physical distancing measures and work from home policies were put into effect, we adapted our methods to include methods for virtual data collection. When using virtual methods (telephone and Zoom) for data collection, the interviewer was potentially unable to view aspects of the participants’ non-verbal communication. Regardless of this, telephone and Zoom have been noted as methods for capturing quality data [48, 49].

## Conclusions

We explored what was working well and challenges experienced by hospital-based care teams in terms of discharge planning for individuals with delayed care transitions. In doing so, we identified three main recommendations to improve team-based functioning for supporting these transitions: (1) improving collaboration with physicians through regular engagement, communication and relationship-building, (2) engaging with frontline providers through co-design and ongoing feedback and (3) partnering across sectors through standardized integrated care approaches. In addressing these recommendations, it is also important for teams to reflect on and build collective competence. Acting on these recommendations has the potential to improve how teams function when supporting care transitions for persons experiencing delays.

## Data Availability

The datasets generated and analyzed in this study are not publicly available to protect the identity of participants. De-identified data cannot be made available unless an ethics amendment is made and approved by all applicable research ethics boards.

## Abbreviation

ALC: Alternate Level of Care
